# Prevalence of intestinal parasitic infections in Brazil: a systematic review

**DOI:** 10.1590/0037-8682-0033-2021

**Published:** 2021-06-02

**Authors:** Ariel Oliveira Celestino, Sarah Cristina Fontes Vieira, Pablo Amercio Silva Lima, Larissa Maria Cardoso Lima Rodrigues, Isabel Ribeiro Santana Lopes, Camila Mendonça França, Ikaro Daniel de Carvalho Barreto, Ricardo Queiroz Gurgel

**Affiliations:** 1 Universidade Federal de Sergipe, Programa de Pós-Graduação em Ciências da Saúde, Aracaju, SE, Brasil.; 2 Universidade Federal de Sergipe, Departamento de Medicina, Aracaju, SE, Brasil.; 3 Universidade Federal de Sergipe, Departamento de Medicina, Campus Lagarto, Lagarto, SE, Brasil.; 4 Universidade Federal Rural de Pernambuco, Programa de Pós-Graduação em Biometria e Estatística Aplicada, Recife, PE, Brasil.

**Keywords:** Parasitic diseases, Health policy, Communicable diseases

## Abstract

**INTRODUCTION:**

Parasitic infections are considered a major public health problem due to their associated morbimortality and negative impact on physical and intellectual development, especially in the at-risk pediatric group. Periodic prophylactic administration of antiparasitic agents against soil-transmitted helminths is recommended by the World Health Organization (WHO) to control parasitic infections and disease burden. We aimed to evaluate the prevalence of intestinal parasitic infections in Brazil.

**METHODS:**

We performed a systematic review by searching the literature found in the PubMed, LILACS, and SciELO databases, followed by a meta-analysis of the proportions from studies published in English, Portuguese, and/or Spanish from January 2000 to May 2018. This systematic review was registered in the PROSPERO database (CRD42018096214).

**RESULTS:**

The prevalence of intestinal parasitic infections (protozoa and/or helminths) in Brazil was 46% (confidence interval: 39-54%), with 99% heterogeneity. Prevalence varied by region: 37%, 51%, 50%, 58%, and 41% in the Southeast, South, Northeast, North, and Central-West regions, respectively. Most studies (32/40) evaluated children (<18 years) and found an average prevalence of 51%. Children also had the highest prevalence in all four regions: Central-West (65%), South (65%), North (58%), Northeast (53%), and Southeast (37%). However, most studies evaluated specific populations, which may have created selection bias. Presumably, this review of intestinal parasitic diseases in Brazil includes the most studies and the largest population ever considered.

**CONCLUSIONS:**

The prevalence of intestinal parasitic infections is high in Brazil, and anthelmintic drugs should be administered periodically as a prophylactic measure, as recommended by the WHO.

## INTRODUCTION

Parasitic diseases are part of the neglected tropical disease group and are considered a major public health problem due to their burden of morbimortality in several population groups. In groups considered at risk, there is a greater burden from these diseases^1^ ^,^ ^2^ ^,^ ^3^.

It is estimated that up to 36% of the world population suffers from some form of parasitosis and up to 55.3% of children, according to the World Health Organization (WHO) in 2016^4^. Common clinical manifestations include diarrhea, reduced absorption of micronutrients, abdominal pain, vomiting, and dehydration^4^. Depending on individual health status, such manifestations can occur with different levels of severity. 

There is a clear association between socioenvironmental factors, such as inadequate sanitation and hygiene levels, and intestinal parasitic infections, as they can promote the ingestion of contaminated water and/or food. Unfortunately, these factors are common in Brazil^5^.

As collecting and testing stool samples prior to treatment is expensive and impractical in many low-income contexts, the WHO recommends prophylactic administration of antiparasitic agents against soil-transmitted helminths for at-risk groups in areas with high prevalence of helminth infections (greater than 20% annually or greater than 50% biannually)^4^ ^,^ ^6^. A program of mass anti-helminthic drug (albendazole) administration has been performed by the Brazilian Ministry of Health (MOH) over the past few years; however, there are no published data on the prevalence of intestinal parasitic infections in large population samples from different regions of Brazil to evaluate the effectiveness of this type of anti-helminthic treatment and whether it should be used, and if so, how often.

It is estimated that the prevalence of intestinal parasitic infections in Brazil is high; however, the rates may be underestimated in clinical practice as not enough patients are tested and the tests themselves have low sensitivity and specificity. Although there have been some studies of specific populations such as children in daycare centers, orphanages, and schools; prisoners; indigenous individuals; residents of *quilombos* (ancient slave settlements); and people who scavenge from rubbish dumps, the representativeness of their data is questionable, as it is likely that the prevalence of parasitic infections is much higher in these groups^1^ ^,^ ^7^ ^-^ ^12^. 

Our study aimed to evaluate the prevalence and characteristics of intestinal parasitic infections in Brazil in order to provide data that can help determine how often the WHO anti-parasitic strategy should be implemented. 

## METHODS

This systematic review of literature was registered in the PROSPERO (International Prospective Register of Systematic Reviews) database (registration code CRD42018096214; http://www.crd.york.ac.uk/PROSPERO). The aim of this study was to answer the research question, “What is the prevalence and characteristics of intestinal parasitic infections in Brazil?” We included cross-sectional observational studies in which the prevalence of intestinal parasitic infections in a Brazilian population was described. The inclusion criteria were observational studies that analyzed human stool samples; were written in Portuguese, English, and/or Spanish; were published between 2000 and 2018; and contained information about location (region, state, city), age, and the number of samples. Studies from which it was impossible to extract data regarding the prevalence of intestinal parasitic infections in Brazil, as well as studies that evaluated other biological samples (not stool), were also excluded.

A search of the literature was conducted using three databases - PubMed (MEDLINE [Medical Literature Analysis and Retrieval System Online]), LILACS (*Literatura Latino-Americana e do Caribe em Ciências da Saúde*), and SciELO (Scientific Electronic Library Online) - in May 2018. The selection of the studies and data extraction were performed by four independent researchers following a predefined protocol, and any disagreements were resolved using a fifth investigator The extracted data included the intestinal parasitic infection prevalence, age of the individuals, region, and diagnostic method.

The search strategy used Medical Subject Headings (MeSH), Health Sciences Descriptors (DeCS), and Boolean operators (OR and AND), which were used to combine descriptors as shown in Table 1. Duplicate references were excluded. The LibreOffice program (The Document Foundation; Berlin, Germany) was used to create spreadsheets, select studies, and extract and describe data in absolute and percentage frequencies. R Software (version 3.6.1; The R Foundation; Vienna, Austria) was used to perform the meta-analysis using a generalized linear mixed model. The prevalence of intestinal parasitic infections by region was calculated using a 95% confidence interval (CI). A funnel plot was used to assess the risk of bias and the I² index to assess the degree of heterogeneity.

**TABLE 1:** Search strategies by database.

Database	Search strategy
PubMed	(parasites[MeSH] OR parasitic diseases[MeSH] OR parasitic infection[MeSH]) AND (prevalence[MeSH] OR epidemiology[MeSH]) AND Brazil[MeSH]
LILACS and SciELO	(parasites OR parasitic diseases) AND (prevalence OR epidemiology) AND Brazil

Abbreviations: **LILACS:** *Literatura Latino-Americana e do Caribe em Ciências da Saúde*; **MeSH:** Medical Subject Headings; **PubMed:** MEDLINE (Medical Literature Analysis and Retrieval System Online); **SciELO:** Scientific Electronic Library Online.

## RESULTS

**FIGURE 1:**
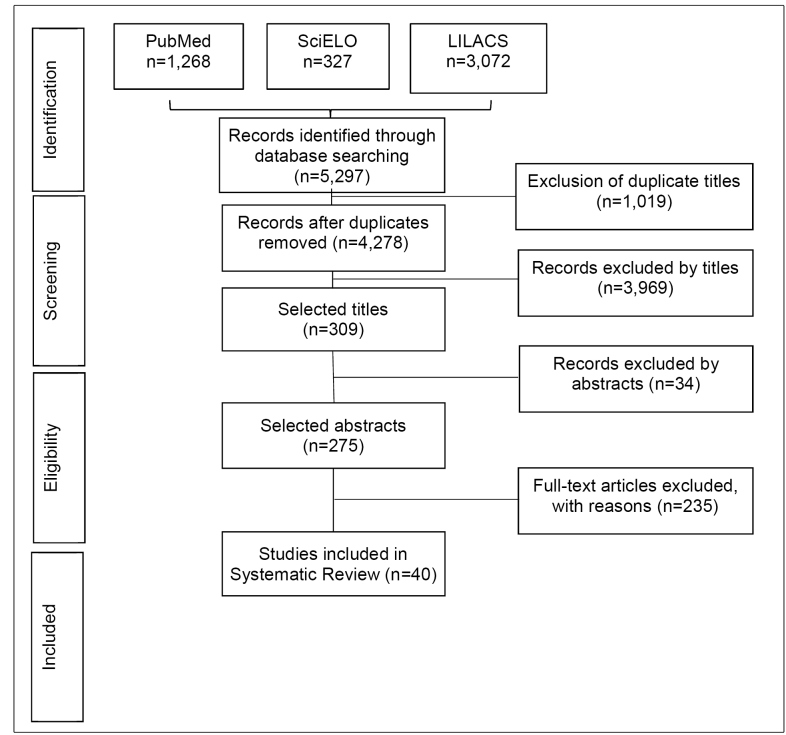
Flow diagram of study search and selection process. Abbreviations: **LILACS:** *Literatura Latino-Americana e do Caribe em Ciências da Saúde*; **PubMed:** MEDLINE (Medical Literature Analysis and Retrieval System Online); **SciELO:** Scientific Electronic Library Online.

A total of 5297 titles were initially identified: 3702 from LILACS, 1268 from PubMed, and 327 from SciELO. Of these, 1019 duplicates were removed. From the remaining 4278 studies, blood sample and molecular studies (n=36) and non-human (n=63), non-epidemiological (n=40), and non-intestinal parasitic (n=3830) studies were removed, leaving 309 studies. Of these, 34 were excluded after reading the abstract, and 235 after reading the full text, leaving 40 studies that met the study criteria and were included in the systematic review (Figure 1). The studies included samples from children, adolescents, adults, and older adults from the five Brazilian regions, and one reported results from two regions (North and Northeast)^13^ (Table 2). Among the selected studies, the most commonly used laboratory test for stool sample analysis was the spontaneous sedimentation method using the Hoffman-Pons-Janner (75%) method, followed by the Kato-Katz technique (22.5%), and the Faust and Cols (20%) method, with some studies using only one of these methods and some using one of these methods in combination with another type of test.

**TABLE 2:** Prevalence of intestinal parasitic infections in selected Brazilian studies according to author, year of publication, region and state, prevalence, and age group

Region	Study	Year	State/Region	N/prevalence (%)	Age range
CENTRAL-WEST	SANTOS *et al.*	2014	DF	193/66.70	Under 18 years
	CURVAL *et al.*	2017	CO	510/20.19	Older than 18 years
	JUNIOR *et al.*	2017	MS	66/43.93	Older than 18 years
NORTH	ORLANDI *et al.*	2006	RO	470/18.20	Under 18 years
	MONTEIRO *et al.*	2009	AM	221/66.40	Under 18 years
	SILVA et al.	2009	AM	123/74.25	Under 18 years
	ESCOBAR *et al.*	2010	AM	245/82.44	Under 18 years
	FONSECA *et al.*	2010	N	1133/38.48	Under 18 years
	BANHOS *et al.*	2017	PA	367/67.57	Under 18 years
NORTHEAST	GUIMARÃES *et al.*	2006	BA	268/30.22	Under 18 years
	BARBOSA *et al.*	2006	PB	11234/45.40	Under 18 years
	SANTOS *et al.*	2006	BA	410/70.73	Under 18 years
	CABRAL *et al.*	2010	BA	348/79.31	Under 18 years
	PALMEIRA *et al.*	2010	AL	481/35.75	Under 18 years
	FONSECA *et al.*	2010	NE	1135/46.64	Under 18 years
	FURTADO *et al.*	2011	PI	294/40.50	Older than 18 years
	SILVA *et al.*	2011	MA	220/53.60	Under 18 years
	CAMPOS *et al.*	2011	RN	86/72.09	Under 18 years
	SOUZA *et al*.	2012	PE	110/48.18	Under 18 years
	FERNANDES *et al.*	2014	PI	251/51.39	Older than 18 years
	SANTOS *et al.*	2017	BA	265/27.16	Older than 18 years
SOUTH	QUADROS *et al.*	2004	SC	200/70.05	Under 18 years
	MARRONE *et al.*	2004	RS	96/82.29	Under 18 years
	BENCKE *et al.*	2006	RS	222/45.94	Under 18 years
	SANTOS *et al.*	2014	SC	57/61.40	Under 18 years
	COLLI *et al*.	2014	PR	150/28.00	Older than 18 years
	SILVA *et al*.	2017	RS	30/12.00	Older than 18 years
	JESKE *et al.*	2017	RS	73/61.64	Older than 18 years
SOUTHEAST	CARVALHO *et al*.	2002	MG	18973/18.06	Under 18 years
	FERREIRA *et al.*	2003	MG	72/59.72	Under 18 years
	FERREIRA *et al*.	2005	SP	902/11.50	Under 18 years
	CARVALHO *et al.*	2006	SP	279/53.40	Under 18 years
	MENEZES *et al.*	2008	MG	472/24.57	Under 18 years
	BARÇANTE *et al*.	2008	MG	176/22.72	Under 18 years
	KORKES *et al.*	2009	SP	120/30.83	Under 18 years
	TASHIMA *et al.*	2009	SP	1000/21.30	Under 18 years
	SILVA *et al.*	2010	MG	161/72.67	Under 18 years
	GONÇALVES *et al.*	2011	MG	133/29.32	Under 18 years
	SANTOS *et al.*	2012	SE	245/51.80	Under 18 years
	BELO *et al*.	2012	MG	1172/28.58	Under 18 years
	FONSECA *et al*.	2017	SP	233/57.50	Under 18 years

Abbreviations: **AL:** Alagoas; **AM:** Amazonas; **BA:** Bahia; **CO:** Central-West ; **DF:** Distrito Federal; **MA:** Maranhão; **MG:** Minas Gerais; **MS:** Mato Grosso do Sul; **N:** North ; **NE:** Northeast ; **PA:** Pará; **PB:** Paraíba; **PE:** Pernambuco, **PI:** Piauí; **PR:** Paraná; **RO:** Rondônia; **RN:** Rio Grande do Norte; **RS:** Rio Grande do Sul; **SC:** Santa Catarina; **SE:** Sergipe; **SP:** São Paulo.

In our review, the population sample was categorized by age groups of over and under 18 years of age. In 32 of the 40 studies (80%), the population samples included individuals under 18 years of age. Sixteen studies provided additional information about population characteristics: daycare children (five studies), schoolchildren (five studies), indigenous people (one study), *quilombolas* (one study), rural areas (one study), orphans (one study), settlement children (one study), and children from a Family Health Program area (one study). Among the eight studies that evaluated individuals over 18 years old, six had additional information about the sample population: food handlers (two studies), waste collectors (two studies), prisoners (one study), and cancer patients (one study).

**FIGURE 2:**
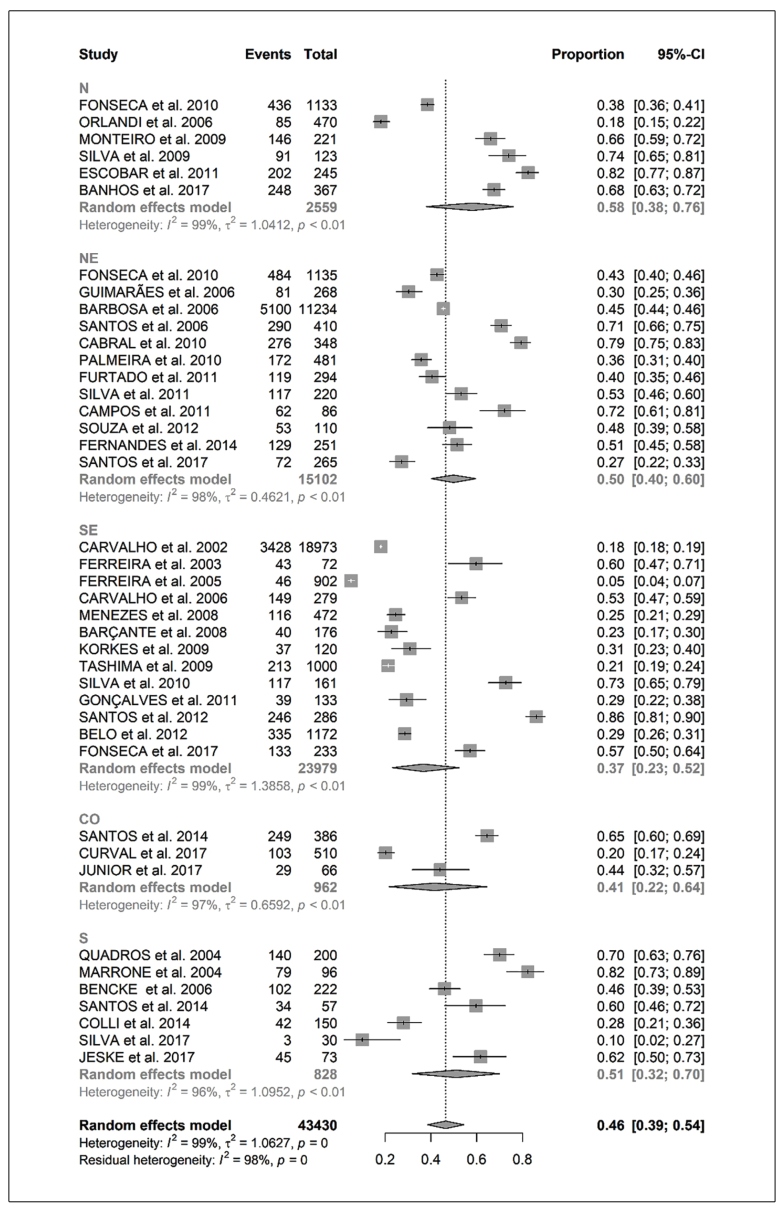
Meta-analysis of proportions of intestinal parasitic infection prevalence by Brazilian region and age groups. Abbreviations: **CI:** confidence interval; **CO:** Central-West region ; **N:** North region; **NE:** Northeast region; **S:** South region; **SE:** Southeast region.

**FIGURE 3:**
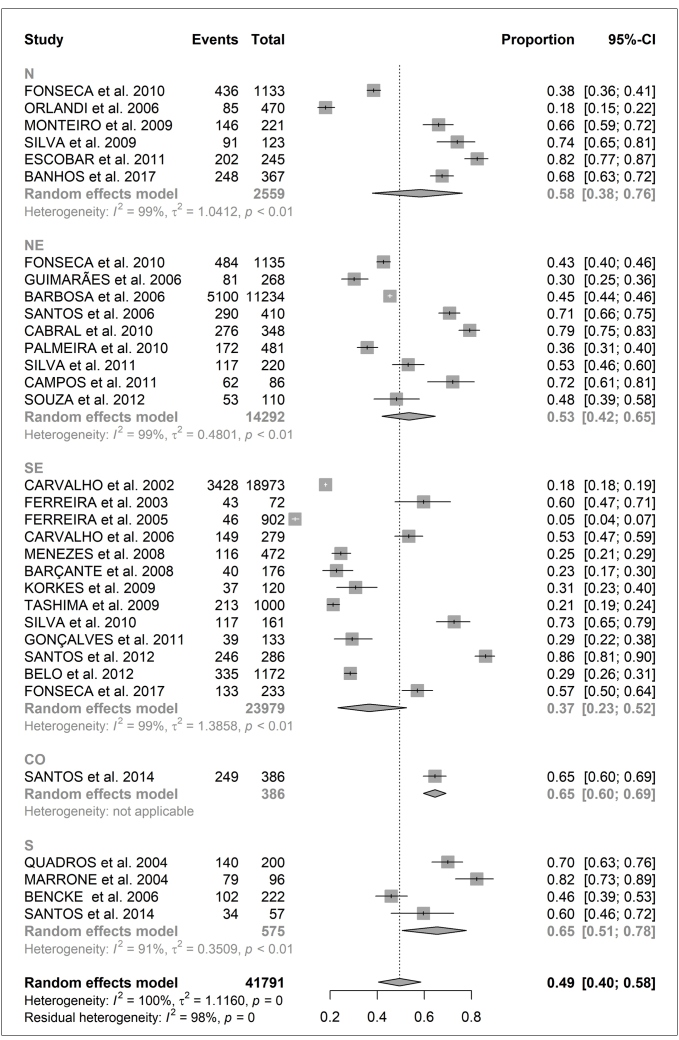
Meta-analysis of proportions of intestinal parasitic infection prevalence among children and adolescents in Brazil. Abbreviations: **CI:** confidence interval; **CO:** Central-West region ; **N:** North region; **NE:** Northeast region; **S:** South region; **SE:** Southeast region.

The meta-analysis of the proportions included in all selected studies showed a prevalence of intestinal parasitic infections of 46% (CI: 39 %-54%) for all of Brazil, regardless of age, with heterogeneity of 99%. Evaluating data by region, we observed a percentage variation from 37% in the Southeast region to 58% in the North region (Figure 2). Considering studies performed in children and adolescents (individuals under 18 years of age), the meta-analysis identified a prevalence of 48% (CI: 40 %-58%) with 99% heterogeneity. In the analysis by region, the prevalence varied from 37% in the Southeast to 65% in the South and Central-West regions (Figure 3).

## DISCUSSION

The WHO recommends prophylactic administration of anti-helminthic drugs for at-risk population groups, including children aged 5-14 years, living in areas with a high prevalence of soil-transmitted helminthic infections^4^. Although there are public policies in place in Brazil to put this recommendation into practice, there is a lack of consistent data on the prevalence of intestinal parasitic infections. 

In our review, most studies (32/40) evaluated the parasitic infection prevalence in children and adolescents. The behavioral and social practices of this age group may explain the higher occurrence of intestinal parasitic infections and puts 5-14-year-olds into the at-risk group. This highlights the importance of controlling parasitic infections in this population to avoid possible complications, including delayed physical and cognitive development, impaired school performance, anemia, and intestinal obstruction, which could be prevented with prophylactic anthelmintic drugs, as recommended by the WHO^2^ ^,^ ^8^ ^,^ ^14^ ^-^ ^17^.

In general, the observed prevalence of intestinal parasitic infections in our study showed high rates of heterogeneity (> 90%) by region. Differences between regions may be explained by the size of the region and the socioeconomic and historical structural disparities that may have affected the prevalence of intestinal parasitic infections^1^ ^,^ ^14^ ^,^ ^18^ ^,^ ^19^. Data from the Brazilian MOH obtained by active screening for infected people from 2005 to 2016 demonstrated a prevalence of soil-transmitted helminthic infections by region similar to that observed in our study^20^. The results from the Northeast (53%), North (58%), Central-West (65%), and South (65%) regions indicated that 50% of intestinal parasitic infections were in children and adolescents, with CIs of 42%-65%, 38-76%, 60%-69%, and 51%-78%, respectively. The high prevalence in the North may result from indigenous habits (hunting, fishing, farming), as most of the samples analyzed in this region belonged to this group. The high prevalence in other regions may be explained by the disorderly growth of some cities, with consequent inadequate basic sanitation, a low human development index, a reduction in quality of life, and poor health care and education, which favors the transmission of diseases, including intestinal parasitosis^13^.

 In the Central-West region, only one study was identified. The sample comprised children and adolescents aged 4-14 years and an intestinal parasitic infection prevalence of 65% was reported. In the South, we observed variations in prevalence data among the different studies. Marrone (2004) reported a high prevalence (82%) in children under five years of age, which far exceeded the average for the region^21^. Bencke (2006) reported a prevalence of 46% in public school students, and Santos (2014) described an intestinal parasitic infection prevalence of 60% among children from daycare centers aged 2-6 years^21^ ^-^ ^24^. There is an increased risk of transmission in environments where children are gathered together, such as in schools^24^. 

In this systematic review, nine studies were conducted in the Northeast region. A meta-analysis of the proportions of general intestinal parasitic infections revealed a prevalence in this region of 53% (CI: 42 %-65%), and most studies (4/9) reported a prevalence of more than 50%. High heterogeneity (99%) was also observed. This finding may be attributed to the characteristics of the studied population, which comprised schoolchildren, residents of *quilombos*, institutionalized children, and individuals from specific communities, rather than samples from the general population^8^ ^,^ ^13^ ^,^ ^25^.

In the Southeast, a meta-analysis of 13 studies showed a prevalence of 35% (CI: 24 %-47%). Most studies did not describe the group characteristics. Ferreira (2003), Carvalho (2006), Silva (2010), Santos (2012), and Fonseca (2017) reported intestinal parasitic infection prevalence of 60%, 53%, 73%, 86%, and 57%, respectively^13^ ^,^ ^25^ ^,^ ^26^ ^,^ ^27^ ^,^ ^28^. These studies were performed with individuals from daycare centers and settlements, where exposure to intestinal parasitic infections was more frequent^26^ ^,^ ^27^ ^,^ ^28^. 

Our study is the first in Brazil to assess the prevalence of intestinal parasitic infections in different regions of the country by performing a meta-analysis of the proportions. It provides important data from 2000 to 2018, which may help in the conception and execution of public policies. We evaluated data from studies whose samples comprised specific populations rather than general population samples, as most of the studies focused on at-risk groups; therefore, data from the general population are limited and have not been published. The heterogeneity of these studies is also a limitation of our study, as it is likely that our calculations overestimated the prevalence of this condition. We highlight that the selected studies reflect the reality of the specific communities that need the most prominent consideration in public health actions, such as the prophylactic administration of anthelmintic drugs. Furthermore, the most commonly used test in the selected studies was the Hoffman method, which is not appropriate for diagnosing parasitic infections that may be not recognized and are not successfully treated with deworming program drugs. However, data from the Brazilian MOH, obtained by active screening from 2005 to 2016, showed a similar distribution by region and reinforces our findings^20^. Another limitation is the lack of studies from the Central-West region, with only one being included, making it impossible to properly evaluate the target age group (5-14 years) in this region with respect to the intervention proposed by the WHO. However, this single study did describe Brazilian children and adolescents of an almost identical age range, and therefore, provides some data representative of this group.

Data from this meta-analysis may not accurately reflect the prevalence of helminths, as it is biased in that it describes data from papers reporting the presence of these parasites, while negative data is often not published. However, we extensively revised the available data so that it can support important decision making in clinical practice and help create public policies that focus on parasitic infection control even with recognized bias. 

Katz (2015) performed a survey to assess the prevalence of schistosomiasis and other intestinal parasitic infections (ancylostomiasis, ascariasis, and trichuriasis) among 197,564 Brazilian school children aged 7-17 years, and reported a prevalence of 0.99% for schistosomiasis, 2.73% for ancylostomiasis, 6% for ascariasis, 5.41% for trichuriasis, and a global prevalence of 15.13% for all parasitic infections^29^. The study considered negative tests and found a lower global prevalence in comparison with our study; however, Katz did not evaluate all parasites and used the Kato-Katz method, which may have impaired the sensitivity of screening for helminths (other than the S. mansoni)^29^.

With respect to parasitic infections, non-governmental organizations, academic institutions, and governments often follow the WHO recommendations for health education activities and periodic mass administration of prophylactic anti-helminthic drugs to at-risk groups living in areas with high prevalence of soil-transmitted helminthic infections. According to the intestinal parasitic infection prevalence rates identified in this review, biannual prophylactic administration of anthelmintic drugs for at-risk groups throughout Brazil is indicated, except in the Southeast region, where annual administration for children and adolescents aged 5-14 years would be sufficient. However, a more effective long-term solution to this problem requires actions that focus on supplying clean drinking water; better sanitation, urban cleaning, and solid waste management; and improved drainage and management of urban rainwater. In the meantime, this deworming strategy needs to be continued in Brazil, especially considering that a significant percentage of the Brazilian population still lives without access to clean water (16.4%) and without sewage collection (46.9%) as reported by the Sanitation Panel Brazil in 2018^30^. The importance of making progress on these socioenvironmental issues is reinforced by the rate of hospitalization for waterborne diseases, which has reached approximately 233,880 hospital admissions per year throughout Brazil^1^ ^,^ ^4^ ^,^ ^14^ ^,^ ^18^ ^,^ ^20^ ^,^ ^30^ ^,^ ^31^. In fact, the high prevalence of parasitic intestinal infections shown in this study highlights the need for improvements in the living conditions of the general population.

It should be highlighted that the clinical benefits of deworming interventions have recently been questioned. A systematic review evaluated their effects on nutritional status, hemoglobin level, cognition, school performance, and survival, but found no evidence of improvement in these indicators^32^. The studies included in that review were from Africa, Asia, and Central America, but none from South America^32^. Our review shows that there is a high prevalence of parasitic infections in Brazil, yet there are no data on the outcomes of deworming interventions.

In conclusion, according to published studies, the prevalence of intestinal parasitic infections in Brazil is high in all five regions of the country, especially in the at-risk populations. The levels of infection found indicate that the country should follow the WHO recommendations for annual or biannual mass administration of prophylactic anthelmintic drugs for children and adolescents aged 5-14 years, depending on the region in which they live. However, further studies evaluating the clinical outcomes of deworming interventions in Brazil are necessary to act as a basis for decision-making on public policies focused on parasitic infection control. The long-term aim should be to improve a range of socioenvironmental factors to provide a lasting solution to this problem.
